# Comparative outcomes in different aortic valve stenosis surgeries and implications of TAVR surgery for cirrhotic patients: A retrospective cohort study

**DOI:** 10.1016/j.amsu.2020.07.056

**Published:** 2020-08-06

**Authors:** Maria Winte, Krysta Contino, Aditi Trivedi, Nikhita Dharbhamulla, John Gaughan, Christopher Deitch, Sangita Phadtare

**Affiliations:** a Cooper Medical School of Rowan University, USA; b Cooper University Health Care, USA

**Keywords:** SAVR, Mini-SVR, TAVR, Cirrhosis

## Abstract

**Background:**

Our hospital system is committed to service to medically underserved, low-income, and minority populations. It is located in a city wherein 37% of people live in poverty. Overall cost effectiveness is part of our patient care quality improvement. Cirrhotic patients are at higher risk for cardiac surgery as cardiopulmonary bypass triggers the release of substances that mimic the physiologic changes seen in cirrhosis. We compared outcomes of surgeries performed for the treatment of aortic valve stenosis, surgical aortic valve replacement (SAVR), mini-surgical valve replacement (mini-SVR), and transcatheter aortic valve replacement (TAVR) with attention to cirrhotic patients.

**Methods:**

This retrospective cohort study looked at the medical records of 457 patients. Demographic data, substance abuse, pre-existing diagnoses, length of stay, outcomes, and lab values were collected for each patient pre- and post-surgery. Fisher's exact test or chi square was used to compare categorical characteristics and outcomes among groups. ANOVA for repeated measures was utilized to compare group differences of continuous measurements over time.

**Results:**

Despite having the highest average age of patients and higher incidence of pre-existing comorbidities, post-operative complications such as arrhythmia, hyponatremia, and coagulopathy developed to a lesser extent in TAVR patients. The length of post-surgery hospital stay was also the least in TAVR patients. TAVR offered better post-operative outcomes in cirrhotic patients as well.

**Conclusions:**

TAVR showed better post-surgical outcomes and provide an option for cardiac surgery for cirrhotic patients. This data will be useful for enabling a patient-centered decision-making process in our population.

## Highlights

• We compared outcomes of aortic valve stenosis surgeries including cirrhotic patients in a medically underserved community.
• Despite the significantly higher age and incidence of comorbidities, TAVR patients showed less post-operative complications.
• TAVR offered better post-operative outcomes in cirrhotic patients as well, making it useful for a patient-centered process.

## 1 Introduction

Our hospital system is committed to service to medically underserved, low-income, and minority populations. Our hospital is located in a city wherein 37% of people live in poverty. The most common ethnic group living below the poverty line in our city is Hispanic while that in the entire county is Caucasian. Consideration of cost effectiveness especially with respect to post-surgery outcomes is one of the aspects of our continuous patient care quality improvement. Aortic stenosis is the most common native valve disease, affecting up to 5% of the elderly population. Surgical aortic valve replacement (SAVR) has been the common surgery used to treat these patients. While SAVR reduces symptoms and improves survival, the surgical risk is significantly increased in patients with co-morbidities and also in elderly patients. Different groups have evaluated the outcomes of transcatheter aortic valve replacement (TAVR) as compared to SAVR in specific populations with somewhat mixed results [[1], [2], [3], [4], [5], [6], [7]].

In this study, we compared outcomes of three types of surgeries performed for treatment of aortic valve stenosis, SAVR, mini-surgical valve replacement (mini-SVR) and TAVR for our patient population. We hypothesized that TAVR patients have decreased intra-operative complications, length of hospital stay, and develop fewer postoperative complications compared to patients who undergo SAVR and mini-SVR, as TAVR is less invasive and can obviate the need for cardiopulmonary bypass. Recently, it has been suggested that in patients who are at increased surgical risk, the decision between SAVR and TAVR should be made by the cardiology team according to individual patient characteristics [8]. We thus evaluated our patient population who had undergone one of these three surgeries with respect to their characteristics and surgical outcomes.

Patients with liver cirrhosis are deemed a high risk population for cardiac surgery and have been noted to have high morbidity and mortality [9]. It has been previously suggested that cardiopulmonary bypass triggers the release of various substances that mimic the physiologic changes seen in cirrhosis such as coagulopathy and other end organ dysfunction. As such, some have hypothesized that avoiding extracorporal circulation may lead to improved outcomes [10]. Other studies have found lower post-procedural complications and mortality when comparing TAVR to SAVR [11]. It has also been found that the 30 day mortality rate in patients with cirrhosis undergoing TAVR was not higher than those without cirrhosis [12]. Our goal in this study was thus also to evaluate surgical outcomes in cirrhotic *versu*s non-cirrhotic patients.

## 2 Methods

The study was approved by the University Health Care Institutional Review Board. A retrospective chart review study was carried out for patients who had undergone SAVR, mini-SVR or TAVR between July 1st, 2012 and June 30th, 2017 (researchregistry5766). Patients under the age of 18 years were excluded from the chart review. International Classification of Diseases, Ninth Revision code (ICD-9) was utilized to identify patients who underwent surgical aortic valve replacement or mini-aortic valve replacement and a departmental database was utilized for identification of patients who had undergone TAVR. In total, we reviewed charts of 457 patients: 117 SAVR patients, 124 mini-SVR patients, and 216 TAVR patients. Demographic data (age, sex, height, weight BMI, ethnic group), substance abuse history, pre-existing diagnoses including liver disease, cardiac comorbidities, renal failure, cancer, cerebral vascular accident, malignancy, psychiatric and rheumatologic diseases, procedural data including date, complications and length of stay were collected for each patient. Information of outcomes collected for each patient included ascites, jaundice, encephalopathy, and variceal bleeding, heart failure, valve failure, arrhythmias, hyponatremia, acute renal failure, bleeding, ongoing myocardial ischemia, hypoalbuminemia, coagulopathy, and death. Various lab values were collected at 30 days pre-operative, the day of the procedure, 30 days post-op, and 90 days post-operative. The diagnosis of cirrhosis was determined by pre-existing diagnosis, imaging findings of cirrhosis on ultrasound, MRI, and CT angiogram (pre-requisite for those undergoing TAVR), and calculation of APRI and FIB-4 scores. APRI score of >0.5 and FIB-4 score of >3.25 were utilized as cut offs to be included in the cirrhotic pool.

### 2.1 Statistical analysis

Fisher's exact test or chi square was used to compare categorical characteristics and outcomes among groups. ANOVA for repeated measures was utilized to compare group differences of continuous measurements over time. Age and body mass index (BMI) were presented as mean. All other characteristics and pre-existing medical conditions were presented as total counts and percentages. Statistical significance was noted for *P* ≤ 0.05.

## 3 Results

We collected data on 457 patients who had undergone SAVR (117), mini-SVR (124) or TAVR (216) in a 5-year period at our University hospital. Demographic data of the patients is presented in Table 1. The average age of the patients who underwent the TAVR (82.7 ± 7.2) was much higher than those who underwent SAVR (65.9 ± 11.9) or mini-SVR (69.5 ± 11.8) procedures. Percentage of females who underwent TAVR was higher (55%) as compared to those who underwent SAVR (44%) mini-SVR (40%). The ethnic breakdown of our patient population revealed that majority were Caucasians (75, 83 and 89% for SAVR, mini-SVR and TAVR procedures, respectively).

**Table 1:** Characteristics of patients undergoing surgery.

Characteristic	SAVR(n = 117)	Mini-SVR(n = 124)	TAVR(n = 216)	P-value
**Age** (years)	65.9 ± 11.9	69.5 ± 11.8	82.7 ± 7.2	<0.001
**BMI**	30.0 ± 7.2	30.5 ± 6.3	27.8 ± 6.2	0.003
**Sex**				0.0220
Male	65 (56%)	75(60%)	99(46%)	
Female	52(44%)	49(40%)	118(55%)	
**Race/ethnicity**				<0.0001
White	88(75%)	103(83%)	192(89%)	
African American	20(17%)	6(5%)	6(3%)	
Hispanic	6(5%)	13(10%)	8(4%)	
Other	3(3%)	2(2%)	11(5%)	
**Alcohol**				
History of alcohol use	55 (47%)	70(56%)	50(23%)	<0.0001
Current alcohol use	51(44%)	65(52%)	44(20%)	<0.0001
**Smoking**				
History of smoking	79 (68%)	66(53%)	123(57%)	0.0542
Currently smokes	21(18%)	11(9%)	7(3%)	<0.0001
**Drug Use**				
History of illicit drug use	7(6%)	6(5%)	2(1%)	0.0132
Current illicit drug use	5(4%)	6(5%)	2(1%)	0.0436

BMI of the TAVR patients was lower on average (27.8) compared to that of SAVR (30) or mini-SVR (30.5) patients. However, considering the average higher age of the TAVR patients, lower BMI values may not necessarily be indicative of lower obesity in this group. Approximately half of the SAVR and mini-SVR patients reported past and current alcohol use, with the percentage being remarkably lower (23%) in the TAVR patients. The data reported on the alcohol use was not quantitative and thus could not allow for conclusions to be drawn regarding alcohol abuse. Patients (53–68%) belonging to all three categories had past smoking history, but that percentage was significantly reduced across the board for current use. The majority of patients denied illicit drug use. Table 2 shows pre-existing medical conditions of the patients undergoing each type of surgery. Hypertension, hyperlipidemia, coronary artery disease, concomitant cardiac disease and atrial fibrillation were observed as pre-existing conditions more signficantly in the patients who underwent TAVR surgery. Psychiatric conditions were more common in SAVR and mini-SVR patients. Overall, the TAVR group had significantly higher age as well as pre-existing comorbidies.

**Table 2:** Pre-existing medical conditions of the patients undergoing surgery.

Pre-existing medical condition	SAVR (N = 117)	Mini-SVR (N = 124)	TAVR (N = 216)	*P* Value
Hypertension	109 (93%)	111(90%)	209(96%)	0.0461
Hyperlipidemia	87(74%)	103(83%)	193(89%)	0.0021
Coronary artery disease	84(72%)	84(68%)	182(84%)	0.0011
Concomitant cardiac disease	55(47%)	31(25%)	146(67%)	<0.0001
Atrial fibrillation	30(26%)	31(25%)	96(44%)	<0.0001
Cancer	22(19%)	31(25%)	65(30%)	0.0797
Chronic kidney disease	26(22%)	15(12%)	29(14%)	0.0852
Stroke	11(9%)	15(12%)	39(18%)	0.0772
Peripheral vascular disease	18(15%)	18(15%)	43(20%)	0.4082
Rheumatic disease	8(7%)	13(11%)	25(12%)	0.3950
Psychiatric conditions	29(25%)	35(28%)	28(13%)	0.0009

Next we compared the post-surgical outcomes in all three groups of patients. The length of the post-surgical hospital stay was 9.06 (±5.02), 7.08 (±3.65) and 6.12 (±5.59) days in SAVR, mini-SVR and TAVR, respectively. Table 3 shows post-surgery complications observed in each group of the patients. We collected 30 day and 90 day post-operative data for each patient. All complications described in Table 3 except death developed within 1–4 days post-surgery, and prior to discharge from the hospital. Death was considered an outcome if it occurred within 1 year of the procedure. Three post-surgical complications were observed to be significantly lower in the TAVR patients namely, arrhythmia (atrial fibrillation) (p < 0.0001), hyponatremia (p = 0.0011), and coagulopathy (p < 0.0001). We found no difference in ongoing myocardial ischemia (OMI) following TAVR, SAVR, or mini-SVR (p = 0.1665). Valve failure was observed more frequently in TAVR patients than SAVR and mini-SVR patients (p = 0.0083); 4% of TAVR patients showed valve failure. Seventeen percent of TAVR patients as compared to the 5–6% SAVR and mini-SVR patients showed hypoalbuminea. One study reported major late bleeding complications (30 days) after TAVR [13]. We did not observe these complications in our TAVR patients.

**Table 3:** Comparison of post-surgical outcomes between SAVR, mini-SVR and TAVR.

Post-surgery complication	SAVR (n = 117)	Mini-SVR (n = 124)	TAVR (n = 216)	*P* Value
Creatinine^a^	9(8%)	8(6%)	19(9%)	0.7944
Renal Failure	23(20%)	14(11%)	35(16%)	0.075
Bleed	7(6%)	2(2%)	13(6%)	0.1559
Congestive heart failure	6(5%)	5(4%)	15(7%)	0.1212
Valve Failure	0(0%)	0(0%)	8(4%)	0.0083
Ongoing myocardial ischemia	1(1%)	0(0%)	4(2%)	0.1665
Arrhythmia	72(62%)	59(48%)	55(25%)	<0.0001
Hyponatremia	45(38%)	39(31%)	44(20%)	0.0011
Hypoalbuminea	6(5%)	7(6%)	36(17%)	0.0002
Coagulopathy	96(82%)	82(66%)	102(47%)	<0.0001
Death	7(6%)	8(6%)	21(10%)	0.3638

a Two times over baseline values.

Our next goal was to compare the three surgical outcomes in cirrhotic *versus* non-cirrhotic patients, however we had only 5 cirrhotic patients who underwent SAVR and only 4 cirrhotic patients who underwent mini-SVR. It was thus not possible to carry out statistically relevant comparison of outcomes within these two surgery groups with respect to existence of cirrhosis (Table 4, Table 5). Out of the 216 patients who underwent TAVR, 49 had cirrhosis and the remaining 167 were non-cirrhotic. We thus carried out statistical analysis of pre-existing conditions and surgical outcomes of non-cirrhotic and cirrhotic TAVR patients. The p values given in Table 4, Table 5 are only for the comparison between TAVR non-cirrhotic and TAVR cirrhotic patients. As seen from Table 4, the pre-existing medical conditions were comparable between these two groups of patients, except for hyperlipidemia, which was higher in TAVR non-cirrhotic patients (p = 0.02). The post-surgical length of stay at the hospital was same in both groups of TAVR patients (6.12 ± 5.59 days). As seen from Table 5, post-surgical outcomes were comparable between the non-cirrhotic and cirrhotic TAVR patients, suggesting that patients with cirrhosis were able to tolerate TAVR surgery well. The only worse post-surgical outcome observed in TAVR cirrhotic patients was congestive heart failure (Table 5).

**Table 4:** Pre-existing medical conditions of non-cirrhotic *versus* cirrhotic surgery patients.

Pre-existing medical condition	SAVR Non-Cirrhotic	SAVR-Cirrhotic	Mini-SVR Non-Cirrhotic	Mini-SVR Cirrhotic	TAVR Non-Cirrhotic	TAVR Cirrhotic	*P* Value^a^ (TAVR patients)
	(n = 112)	(n = 5)	(n = 120)	(n = 4)	(n = 167)	(n = 49)	
Hypertension	104(93%)	5(100%)	107(89%)	4(100)	159(95%)	49(100%)	0.20
Hyperlipidemia	84(75%)	3(60%)	100(83%)	3(75)	153(92%)	39(80%)	0.02
Coronary artery disease	79(71%)	5(100%)	82(68%)	2(50)	137(82%)	44(90%)	0.27
Concomitant cardiac disease	52(46%)	3(60%)	30(25%)	1(25)	111(66%)	34(69%)	0.73
Atrial fibrillation	28(25%)	2(40%)	29(24%)	2(50)	73(44%)	23(47%)	0.62
Cancer	22(20%)	0(0%)	29 (24%)	2(50)	47(28%)	18(37%)	0.29
Chronic kidney disease	24(21%)	2(40%)	15 (13%)	0(0)	19(11%)	10(20%)	0.10
Stroke	11(10%)	0(0%)	14 (12%)	1(25)	33(20%)	6(12%)	0.29
Peripheral vascular disease	17(15%)	1(20%)	18 (15%)	0(0)	34(20%)	9(18%)	0.84
Rheumatic disease	8(7%)	0(0%)	13 (11%)	0(0)	21(13%)	4(8%)	0.61
Psychiatric disease	29(26%)	0(0%)	35 (29%)	0(0)	22(13%)	6(12%)	1.00

a P values are given only for the comparision between TAVR non-cirrhotic and TAVR cirrhotic patients.

**Table 5:** Comparison of post-surgical outcomes between non-cirrhotic and cirrhotic surgery patients.

Post-surgery complication	SAVR Non-Cirrhotic	SAVR-Cirrhotic	Mini-SVR Non-Cirrhotic	Mini - SVR-Cirrhotic	TAVR Non-Cirrhotic	TAVR-Cirrhotic	*P* Value* (TAVR patients)
	(N = 112)	(N = 5)	(n = 120)	(n = 4)	(n = 167)	(n = 49)	
Creatinine^a^	9(8%)	0(0%)	8(7%)	0(0%)	13(8%)	6(12%)	0.2683
Renal Failure	21(19%)	2(40%)	14(12%)	0(0%)	23(14%)	12(24%)	0.0912
Bleed	7(6%)	0(0%)	2(2%)	0(0%)	8(5%)	5(10%)	0.0974
Congestive heart failure	6(5%)	0(0%)	5(4%)	0(0%)	9(5%)	6(12%)	0.008
Valve Failure	0(0%)	0(0%)	0(0%)	0(0%)	5(3%)	3(6%)	0.2598
Ongoing myocardial ischemia	1(1%)	0(0%)	0(0%)	0(0%)	3(2%)	1(2%)	0.7951
Arrhythmia	68(61%)	4(80%)	55(46%)	4(100%)	41(25%)	14(29%)	0.5782
Hyponatremia	41(37%)	4(80%)	38(32%)	1(25%)	31(19%)	13(27%)	0.2412
Hypoalbuminea	6(5%)	0(0%)	7(6%)	0(0%)	25(15%)	11(22%)	0.5089
Coagulopathy	92(82%)	4(80%)	78(65%)	4(100%)	78(47%)	23(47%)	0.8186
Death	6(5%)	1(20%)	8(7%)	0(0%)	15(9%)	6(12%)	0.4411

*P values are given only for the comparision between TAVR non-cirrhotic and TAVR cirrhotic patients.

a Two times over baseline values.

## 4 Discussion

Our data showed that TAVR patients had better post-surgical outcomes compared to SAVR and mini-SVR. This is especially noteworthy as the TAVR group of patients was significantly older and had higher incidence of pre-existing comorbidities. This is consistent with the report that TAVR may be a better option in elderly patients with comorbidities as it allows implantation of a prosthetic heart valve within the diseased native aortic valve without the need for open heart surgery and cardiopulmonary bypass [14]. As mentioned above, the average age of our patients who underwent the TAVR surgery was much higher than those who underwent SAVR or mini-SVR procedures. Recently, however there have been concerns about the use of TAVR especially in younger patients, who are otherwise excellent candidates for SAVR due to the unknown long-term outcomes of the TAVR procedure, such as neurological damage. The many under-studied complications and unknown long-term outcomes of the TAVR procedure thus lead to cautionary note for this use of this procedure in low-risk patients [15,16].

It was observed that post-operative complications from cardiac surgery are higher in patients with liver disease increasing their surgical risk such as bleeding complications, post-operative worsening of liver disease and higher incidence of death. Cardiopulmonary bypass is not well tolerated in cirrhotic patients due to possible release of vasoactive substances caused by the bypass [17]. Studies showed that it is possible to achieve lower rates of liver decompensation with TAVR surgery. Arrhythmia, a common and dangerous consequence of cardiac surgery, was decreased with TAVR, making it a superior option for the treatment of aortic valve pathology in cirrhotic patients as well. Cirrhotic and end-stage liver patients are more likely to get general complications and physiological changes in addition to varices, ascites, and portal hypertension. These can make them prone to coagulopathy, infections, and organ dysfunctions [11]. Notably, coagulopathy was a significantly lower post-surgical complication in both of our cirrhotic and non-cirrhotic TAVR patients as compared to SAVR and mini-SVR patients (Table 3, Table 5). Our study had a limitation in that we did not have sufficient number of cirrhotic patients undergoing SAVR and mini-SVR surgeries. Thus a detail statistical comparison with the cirrhotic TAVR patients could not be carried out. Further studies are needed to compare TAVR with SAVR and mini-SVR in cirrhotic patients.

There is difference of opinion about the risk of mortality of TAVR in more advanced cirrhosis. Some studies suggested that for patients with more advanced cirrhosis, the risk of mortality is very high [17,18], while another study showed that advanced cirrhosis should not exclude patients from undergoing TAVR surgery [19].

Although procedural costs are higher with TAVR than SAVR, it is argued that total cost differences for the index hospitalization are marginally higher owing to reductions in length of stay with TAVR. Follow-up costs are also significantly lower with TAVR than with SAVR. Over a lifetime timeframe, TAVR was thus projected to lower total costs by $8000 to $10,000 [20]. However, there is no uniform consensus on this point as the future of TAVR prostheses remains unknown beyond the short term [15]. One of the limitations of our study is that we did not carry out case-based cost calculations to determine cost-effectiveness of TAVR in our patient population.

## 5 Conclusion

Our data shows that TAVR offers comparable if not superior post-surgical outcomes when compared to current treatment options for aortic valve pathology. It also supports the notion that liver cirrhosis should not preclude patients from cardiac surgery. Although we have not carried out case-based cost studies, considerations of procedural and follow up costs suggest that TAVR may be a cost-effective option for patients, especially based on the average age and pre-existing comorbidities of patients who underwent this procedure. Our study provides a step toward enabling a patient-centered decision-making process in our medically underserved and low-income patient population.

## Provenance and peer review

Not commissioned, externally peer reviewed.

## Ethical approval

This retrospective study was reviewed and approved by the Institutional Review Board of Cooper Health System on 09/18/2017 (Reference number 17-132EX).This study has a waiver of consent and HIPAA authorization.

## Sources of funding

None.

## Author contribution

Maria Winte: data collection, data processing, reviewing. Krysta Contino: data collection, writing, reviewing. Aditi Trivedi: data collection. Nikhita Dharbhamulla: data collection. John Gaughan,: statistical analysis. Christopher Deitch: study concept, study design, reviewing. Sangita Phadtare: literature review, study concept, study design, writing, reviewing.

## Research registration number

Name of the registry: ResearchRegistry

Unique Identifying number or registration ID: researchregistry5766.

Hyperlink to your specific registration (must be publicly accessible and will be checked): https://www.researchregistry.com/browse-the-registry#home/registrationdetails/5efa0dd97103e90016268c5f/

## Guarantor

Sangita Phadtare and Krysta Contino.

## Consent

This is a retrospective cohort study based on electronic medical charts review. This study has a waiver of consent and HIPAA authorization. This retrospective study was reviewed and approved by the Institutional Review Board of Cooper Health System on 09/18/2017 (Reference number 17-132EX).

## Declaration of competing interest

No conflicts of interest to declare.

## References

[1] Adams D.H., Popma J.J., Reardon M.J. (2014). Transcatheter aortic-valve replacement with a self-expanding prosthesis. N. Engl. J. Med..

[2] Kodali S.K., Williams M.R., Smith C.R., Svensson L.G., Webb J.G., Makkar R.R., Fontana G.P., Dewey T.M., Thourani V.H., Pichard A.D. (2012). Two-year outcomes after transcatheter or surgical aortic-valve replacement. N. Engl. J. Med..

[3] Reardon M.J., Adams D.H., Kleiman N.S., Yakubov S.J., Coselli J.S., Deeb G.M., Gleason T.G., Lee J.S., Hermiller J.B., Chetcuti S. (2015). 2-Year outcomes in patients undergoing surgical or self-expanding transcatheter aortic valve replacement. J. Am. Coll. Cardiol..

[4] Kumbhani D.J., Banerjee S. (2016). 3-Year results of a TAVR trial in high surgical risk patients. J. Am. Coll. Cardiol..

[5] Deeb G.M., Reardon M.J., Chetcuti S., Patel H.J., Grossman P.M., Yakubov S.J., Kleiman N.S., Coselli J.S., Gleason T.G., Lee J.S. (2016). 3-Year outcomes in high-risk patients who underwent surgical or transcatheter aortic valve replacement. J. Am. Coll. Cardiol..

[6] Mack M.J., Leon M.B., Smith C.R., Miller D.C., Moses J.W., Tuzcu E.M., Webb J.G., Douglas P.S., Anderson W.N., Blackstone E.H. (2015). 5-year outcomes of transcatheter aortic valve replacement or surgical aortic valve replacement for high surgical risk patients with aortic stenosis (PARTNER 1): a randomised controlled trial. Lancet.

[7] Moat N.E., Ludman P., de Belder M.A., Bridgewater B., Cunningham A.D., Young C.P., Thomas M., Kovac J., Spyt T., MacCarthy P.A. (2011). Long-term outcomes after transcatheter aortic valve implantation in high-risk patients with severe aortic stenosis: the U.K. TAVI (United Kingdom Transcatheter Aortic Valve Implantation) Registry. J. Am. Coll. Cardiol..

[8] Paparella D., Santarpino G., Malvindi P.G., Moscarelli M., Marchese A., Guida P., Carbone C., Gregorini R., Martinelli L., Comoglio C. (2019). Minimally invasive surgical versus transcatheter aortic valve replacement: a multicenter study. IJC *Heart & Vasculature*.

[9] Jacob K.A., Hjortnaes J., Kranenburg G., de Heer F., Kluin J. (2015). Mortality after cardiac surgery in patients with liver cirrhosis classified by the Child-Pugh score. Interact. Cardiovasc. Thorac. Surg..

[10] Greason K.L., Mathew V., Wiesner R.H., Suri R.M., Rihal C.S. (2013). Transcatheter aortic valve replacement in patients with cirrhosis. J. Cardio. Surg..

[11] Thakkar B., Patel A., Mohamad B., Patel N.J., Bhatt P., Bhimani R., Arora S., Savani C., Solanki S., Sonani R. (2016). Transcatheter aortic valve replacement versus surgical aortic valve replacement in patients with cirrhosis. Cathet. Cardiovasc. Interv..

[12] Wendt D., Kahlert P., Canbay A., Knipp A.S., Thoenes M., Cremer G., Al-Rashid F., Janosi R.A., El-Chilali K., Kamler M. (2017). Impact of liver indicators on clinical outcome in patients undergoing transcatheter aortic valve implantation. Ann. Thorac. Surg..

[13] Genereux P., Cohen D.J., Mack M., Rodes-Cabau M.J., Yadav M., Xu K., Parvataneni R., Hahn R., Kodali S.K., Webb J.G. (2014). Incidence, predictors, and prognostic impact of late bleeding complications after transcatheter aortic valve replacement. J. Am. Coll. Cardiol..

[14] Siqueira D., Abizaid A., Arrais M., Sousa J.E. (2012). Transcatheter aortic valve replacement in elderly patients. J. Geri. Ccardiol..

[15] Samarendra P. (2020). A precipitous decision: transcatheter aortic valve replacement in low-risk patients. Cardiol. Res..

[16] Ielasi A., Latib A., Tespili M., Donatelli F. (2019). Current results and remaining challenges of trans-catheter aortic valve replacement expansion in intermediate and low risk patients. IJC Heart & Vasculature.

[17] Shah A.M., Ogbara J., Herrmann H.C., Fox Z., Kadakia M., Anwaruddin S., Bavaria J.E., Desai N.D., Jagasia D., Szeto W.Y. (2015). Outcomes of transcatheter aortic valve replacement in patients with chronic liver disease. Cathet. Cardiovasc. Interv..

[18] Modi A., Vohra H.A., Barlow C.W. (2010). Do patients with liver cirrhosis undergoing cardiac surgery have acceptable outcomes?. Interact. Cardiovasc. Thorac. Surg..

[19] Lin C.H., Hsu R.B. (2014). Cardiac surgery in patients with liver cirrhosis: risk factors for predicting mortality. World J. Gastroenterol..

[20] Baron S.J., Wang K., Arnold S.V., Magnuson E.A., Whisenant B., Brieke A., Rinaldi M., Asgar A.W., Lindenfeld J., Abraham W.T. (2019). Cost-effectiveness of transcatheter mitral valve repair versus medical therapy in patients with heart failure and secondary mitral regurgitation: results from the COAPT trial. Circulation.

